# Nitric Oxide Has a Concentration-Dependent Effect on the Cell Cycle Acting *via* EIN2 in *Arabidopsis thaliana* Cultured Cells

**DOI:** 10.3389/fphys.2017.00142

**Published:** 2017-03-10

**Authors:** Galina V. Novikova, Luis A. J. Mur, Alexander V. Nosov, Artem A. Fomenkov, Kirill S. Mironov, Anna S. Mamaeva, Evgeny S. Shilov, Victor Y. Rakitin, Michael A. Hall

**Affiliations:** ^1^Laboratory of Intracellular Regulation, K.A. Timiryazev Institute of Plant Physiology, Russian Academy of SciencesMoscow, Russia; ^2^Molecular Plant Pathology Group, Institute of Biological, Environmental and Rural Sciences, Aberystwyth UniversityAberystwyth, UK; ^3^Department of Immunology, M.V. Lomonosov Moscow State UniversityMoscow, Russia

**Keywords:** *Arabidopsis thaliana*, cell culture, cell cycle, cell proliferation, ethylene, nitric oxide

## Abstract

Ethylene is known to influence the cell cycle (CC) via poorly characterized roles whilst nitric oxide (NO) has well-established roles in the animal CC but analogous role(s) have not been reported for plants. As NO and ethylene signaling events often interact we examined their role in CC in cultured cells derived from *Arabidopsis thaliana* wild-type (Col-0) plants and from ethylene-insensitive mutant *ein2-1* plants. Both NO and ethylene were produced mainly during the first 5 days of the sub-cultivation period corresponding to the period of active cell division. However, in *ein2-1* cells, ethylene generation was significantly reduced while NO levels were increased. With application of a range of concentrations of the NO donor, sodium nitroprusside (SNP) (between 20 and 500 μM) ethylene production was significantly diminished in Col-0 but unchanged in *ein2-1* cells. Flow cytometry assays showed that in Col-0 cells treatments with 5 and 10 μM SNP concentrations led to an increase in S-phase cell number indicating the stimulation of G1/S transition. However, at ≥20 μM SNP CC progression was restrained at G1/S transition. In the mutant *ein2-1* strain, the index of S-phase cells was not altered at 5–10 μM SNP but decreased dramatically at higher SNP concentrations. Concomitantly, 5 μM SNP induced transcription of genes encoding *CDKA;1* and *CYCD3;1* in Col-0 cells whereas transcription of *CDK*s and *CYC*s were not significantly altered in *ein2-1* cells at any SNP concentrations examined. Hence, it is appears that EIN2 is required for full responses at each SNP concentration. In *ein2-1* cells, greater amounts of NO, reactive oxygen species, and the tyrosine-nitrating peroxynitrite radical were detected, possibly indicating NO-dependent post-translational protein modifications which could stop CC. Thus, we suggest that in *Arabidopsis* cultured cells NO affects CC progression as a concentration-dependent modulator with a dependency on EIN2 for both ethylene production and a NO/ethylene regulatory function.

## Introduction

The cell cycle (CC) is one of the most conserved processes operating in eukaryotic cells and its integrity is essential for an organism's shape and function. The CC has been extensively described in both plants (Dewitte and Murray, 2003) and animals (Norbury and Nurse, 1992) and is regulated by the sequential expression of cyclin-dependent kinases (CDK) which are activated by binding to the cyclins (CYC) (Dorée and Galas, 1994). However, newly emerging CC regulatory steps, especially in plants, require further definition. In particular, ethylene can positively or negatively affect CC progression but these steps have been poorly defined. For example, Love et al. (2009) used both the transgenic ethylene-insensitive and ethylene-overproducing hybrid aspen (*Populus tremula* × *tremuloides*) and treatment with an ethylene perception inhibitor 1-methylcyclopropene (1-MCP) to demonstrate that ethylene stimulates cell division in the cambial meristem. Similarly, Ortega-Martínez et al. (2007) suggested that ethylene production and ethylene-modulated cell division was suppressed in the quiescent center of *Arabidopsis thaliana* roots. On the other hand, CC arrest in parallel with an increase in 1-aminocyclopropane-1-carboxylate (ACC) levels and the activation of ethylene signaling in *A. thaliana* leaves was observed during osmotic stress (Skirycz et al., 2011).

Another unknown in the plant CC is the role of nitric oxide (NO). There is a considerable literature describing the biological role(s) of NO in plants such as seed dormancy, growth, and development, senescence, respiration, photosynthesis, programmed cell death, antioxidant defense system (for review, Hayat et al., 2009) with the most detailed information available for NO effects during biotic stress (for reviews, Delledonne et al., 1998; Astier and Lindermayr, 2012; Mur et al., 2013) but the potential role of NO in regulating plant CC remains to be defined. This is the case although many studies in mammalian cells have demonstrated the importance of NO to CC progression (Takagi et al., 1994; Tanner et al., 2000; Cui et al., 2005; Kumar et al., 2010).

Preliminary evidence is suggestive of a role for NO in influencing plant CC. Ötvös et al. (2005) studied NO effects on protoplasts derived from alfalfa leaves and showed that low concentrations of chemical NO-donors stimulated incorporation of 5-bromo-2′-deoxyuridine (BrdU), i.e., initiated DNA synthesis. However, higher NO-donor concentrations blocked DNA synthesis. Based on these results it was first speculated that NO at low concentrations positively affected G1/S transition. Similar, concentration-dependent effects for NO, were also suggested by Bai et al. (2012) based on an *Arabidopsis* apical root meristem model using the NO-donor, sodium nitroprusside (SNP) (2–50 μM). SNP inhibited the main root growth at concentrations higher than 20 μM and its effect was accompanied by a decrease in the size of the root apical meristem, reduced number of cells expressing mitotic cyclin B1;1 (*CYCB1;1*) and the appearance of cells with damaged DNA. No concentrations of SNP reduced the number of 2C DNA nuclei further indicating that SNP blocked G1/S transition. Considering endogenously produced NO, application of the NO scavenger 2-(4-carboxyphenyl)-4, 4, 5, 5-tetramethylimidazoline-1-oxyl-3-oxide (cPTIO) or the use of the *atnoa1* mutant (with lower NO production), resulted in reduced *CYCD3;1* expression following the application of *trans*-zeatin (Shen et al., 2013). There was also an increase in the number of nuclei with 4 and 8C DNA in *atnoa1* compared to wild-type *Arabidopsis* which lead the authors to suggest that NO inhibits endoreduplication and stimulates G1/S transition. In line with this, Zhu et al. (2016) demonstrated NO-induced accumulation of cells in the S-phase during adventitious root formation in cucumber due to up-regulation of the genes involved in G1/S transition, *CYCA, CYCB, CDKA*, and *CDKB*. Other studies suggested that NO can act at G0 phase. Addition of cPTIO blocked the derivation of tomato lateral root primordia from the pericycle while applying SNP restored this process by increasing the expression of *CYCD3;1* and reducing the expression of *KRP2*—a negative regulator of the CC (Correa-Aragunde et al., 2006).

In our previous studies on biotic stress, we have demonstrated that NO can stimulate ethylene production in tobacco and *Arabidopsis* (Mur et al., 2008, 2012). This is also the case with somatic embryogenesis where increasing NO production through suppressing the expression of NO oxidizing Glb1 (hemoglobin class 1) also increased the generation of ethylene (Mira et al., 2015). However, with fruit ripening (Manjunatha et al., 2012), abscission of plant organs (Para-Lobato and Gomez-Jimenes, 2011), and the regulation of leaf senescence (Mishina et al., 2007) NO counteracts ethylene production. Niu and Guo (2012) used both *Atnoa1* and the *ethylene insensitive 2 (ein2-1)* mutants to suggest that NO regulates dark-induced leaf senescence through EIN2. Endoplasmic reticulum (ER)-localized EIN2 (Bisson et al., 2009) is a positive regulator in ethylene signaling in *Arabidopsis* and *ein2* mutants plants are insensitive to almost all aspects of ethylene responses (Alonso et al., 1999). According to the canonical ethylene signaling pathway, in the absence of ethylene, the ethylene receptors at ER interact with CONSTITUTIVE TRIPLE RESPONSE 1 (CTR1) protein kinase, which represses EIN2 followed by ETHYLENE INSENSITIVE 3 (EIN3) and EIN-LIKE 1 (EIL1). These latter are two transcription factors that control the majority of ethylene responses (Chang et al., 2013). Without ethylene, EIN3 and EIL1 are degraded through the action of F-box proteins EIN3-BINDING F-BOX 1 (EBF1) and EBF 2 (Guo and Ecker, 2003), whereas ethylene decreases the reduction of EBF1/2 protein levels in an EIN2-dependent manner. Thus, EIN3/EIL1 are stabilized and activate ethylene responses (An et al., 2010). EIN3/EIL1 also directly regulate the expression of a diverse array of genes including *ERF1* (*ETHYLENE RESPONSE FACTOR 1*) (Chang et al., 2013).

In this current study we sought not only to define NO and ethylene-mediated effects on the plant CC but possible interactions between these signals. We chose to exploit cultured *Arabidopsis* cells as this would avoid problems associated with intact plants where CC progression may be masked by the presence of diverse intercellular interactions and complex developmental programs. Thus, we studied NO effects on the CC of cultured wild-type (Col-0) and *ein2-1 Arabidopsis* cells. Here we demonstrate that NO acts as a concentration-dependent modulator of CC progression with a dependency on EIN2 for both of ethylene production and a NO/ethylene regulatory functions.

## Materials and methods

### Derivation of suspension cell cultures

Suspension cell cultures of *Arabidopsis thaliana* (L.) Heynh. wild type (Col-0) and the ethylene-insensitive mutant *ein2-1* were generated by A.V. Nosov and A.A. Fomenkov and deposited at the All-Russia Collection of Cultivated Cells of Higher Plants as NFC-0 (for Col-0) and NFCE-2 (for *ein2-1*). Both cell strains were cultured in 50 mL of Schenk and Hildebrandt medium (Schenk and Hildebrandt, 1972) supplemented with 3% sucrose, 1 mg L^−1^ 2,4-D (Sigma, St. Louis, Missouri, USA) and 0.1 mg L^−1^ kinetin (Sigma). Cells were grown under constant agitation (110 rpm) at 26°C and 70% humidity. The time of sub-culture was 10 days and inoculated volumes were 1 mL for Col-0 and 2.5 mL for *ein2-1*. To confirm that the *ein2-1* mutation is retained in the cultured cells allele-specific PCR was performed periodically as described by Stepanchenko et al. (2012).

### Treatments with sodium nitroprusside (SNP)

Cells were treated with freshly prepared solutions of the NO-donor sodium nitroprusside (SNP, Sigma), which were added to the culture medium at concentrations ranging from 2 to 10,000 μM. Cells were incubated with the NO-donor in the light (140 μM m^−2^ s^−1^) on an orbital shaker (110 rpm) at 26°C. The incubation time was chosen based on the kinetics of SNP decomposition in the solution (Floryszak-Wieczorek et al., 2006). In all experiments, “NO-exhausted” SNP solutions (generated by allowing NO release in the light for 48 h prior to use) were used as a control treatment. To demonstrate that SNP effects were specific to NO, cultured cells were treated with 100 μM of the NO scavenger cPTIO prior SNP application. All experiments were performed on the fourth day after inoculation of cells into fresh medium when Col-0 and *ein2-1* cells were dividing actively.

### Determination of NO, ONOO^−^, and ROS production in Col-0 and *ein2-1* cells

NO production was assessed in 1 mL of cell suspensions with 5 μM DAF-FM DA (4-amino-5-methylamino-2′, 7′-difluorofluorescein diacetate), peroxynitrite (ONOO^−^)—with 5 μM APF (aminophenyl fluorescein), superoxide with 10 μM DHE (dihydroethidium), intracellular ROS (ROS_in_)—with 1 μM DCFH-DA (dichlorodihydrofluorescein diacetate). All dyes were purchased from Sigma.

Cells were incubated with the corresponding fluorescent dye for 15 min in the dark and then 200 μL aliquots of cells were transferred to 96-well flat bottom plates (Greiner Bio One, Germany). Plates were scanned in Typhoon Trio^+^ Imager (GE Healthcare, Boston, Massachusetts, USA) at λ_ex_ 480 nm and λ_em_ BP 520 nm/40 nm for DAF-FM DA, DCFH-DA, and APF, and λ_ex_ 480 nm and λ_em_ BP 580 nm/30 nm for DHE.

NO levels during the sub-cultivation period were expressed as arbitrary units (AU) per 1 g of cell dry weight (DW). Values for ONOO^−^, superoxide and ROS_in_ production calculated as fluorescence units per 1 μg of protein measured with a BCA Protein Assay Kit (Sigma).

Dye fluorescence was also imaged using an Axio Imager Z2 microscope (Carl Zeiss, Oberkochen, Germany) with a digital camera AxioCam MR, and the appropriate filter units were used. DAF-FM DA was detected at λ_ex_ BP 475 nm/40 nm; λ_em_ BP 530 nm/50 nm. For continuous monitoring of DAF-FM DA fluorescence, module ApoTome (Carl Zeiss) was used. The images were processed using the program AxioVision 4.8 (Carl Zeiss).

All fluorescence experiments were performed with at least three independent sets of Col-0 and *ein2-1* cells. Data presented are means of three independent experiments with five replicates ± SE (standard errors).

### Measurement of ethylene and O_2_ levels in Col-0 and *ein2-1* cells

Ethylene and O_2_ levels in cultured cells were measured as described by Rakitin and Rakitin (1986). Briefly, 3–5 mL of cell suspensions in 15 mL glass vials were mounted on an orbital shaker (110 rpm) and kept at 26°C for 10 min in the dark, then vials were sealed with Suba Seals (Sigma) and kept at the same conditions for 0.5–1 h. The vials with fresh SH medium only were used as a blank reference. Prior to measurement, ethylene was concentrated in a Porapak N column (80–100 mesh, 70 × 4 mm, Supelko, purchased from Sigma) at −30°C. After desorption at 50°C, ethylene was measured with a gas chromatograph fitted with a Poropak N column (80–100 mesh, 3 m × 2 mm, Supelko). Ethylene production was calculated as nL per hour per 1 g DW. O_2_ content was estimated by gas absorption chromatography.

### Determination of the viability of SNP-treated Col-0 and *ein2-1* cells

Cell viability was assessed after 24 h treatment of Col-0 and *ein2-1* cells with SNP at concentrations from 100 to 10,000 μM. Cells were stained with a 0.02% aqueous solution of Erythrosin B (Sigma) and counted using a Univar microscope (Reichert-Jung, Austria). Viability was calculated as percentage of the number of cells unstained with Erythrosin B from their total number.

### Isolation of protoplasts from Col-0 and *ein2-1* cultured cells

Protoplasts from both strains of cultured cells were isolated at 26°C on an orbital shaker (120 rpm) within 1.0–1.5 h, as described earlier (Nosov et al., 2014). Aliquots (5 mL) of Col-0 and *ein2-1* cell suspensions were mixed with equal volumes of solution with major inorganic components of SH medium containing 0.8 M sorbitol, 8 mM CaCl_2_, 25 mM MES at pH 5.7, 2% cellulase Onozuka R10 (Kinki Yakult, Tokyo, Japan), 0.3% pectinase Macerozyme R10 (Kinki Yakult), and 0.8% hemicellulase Driselase (Fluka, Seelze, Germany). After 1.0–1.5 h incubation, the suspensions were filtered through nylon mesh (40 μm pore size) and washed twice with 2.5 mM CaCl_2_ in 0.5 M sorbitol. For cell number counting, the protoplast suspensions in the enzyme solution were used without additional filtration and washing. For flow cytometry analysis and microscopy, 1.5 mL of protoplast suspension was added drop-wise into 3.5 mL of cold (4°C) methanol. Protoplasts were fixed in methanol and stored at 4°C before use.

### Determination of S-phase cells by flow cytometry

Cells in S-phase of the CC were determined by incorporation of 5-ethynyl-2′-deoxyuridine (EdU) into DNA. EdU (Invitrogen, Carlsbad, California, USA) was added to a final concentration of 20 μM. After 1-h incubation with EdU its incorporation into DNA was terminated by the addition of 200 μM 2′-deoxythymidine (Sigma) for 5 min then the protoplasts were isolated as described above.

Methanol-fixed protoplasts were washed twice in 0.5 M sorbitol without CaCl_2_, once in phosphate-buffered saline (PBS, Sigma) with 0.1% Triton X-100 (Sigma), and once in PBS without Triton X-100. EdU incorporation into newly-synthesized DNA was detected by reaction with Alexa Fluor 488 using Click-iT EdU Alexa Fluor 488 HCS Kit (Invitrogen), while DNA was stained with 100 ng mL^−1^ DAPI (4′, 6-diamidino-2-phenylindol, Sigma). On average, 100,000 protoplasts were analyzed with a Gallios flow cytometer (Beckman Coulter, Brea, California, USA). Alexa Fluor 488 fluorescence was detected in channel FL1 (λ_ex_ 488 nm, λ_em_ 505–545 nm), whereas channel FL9 (λ_ex_ 405 nm, λ_em_ 430–470 nm) was adopted for reading DAPI fluorescence. Data analysis was performed using FlowJo 7.6.2 (http://www.flowjo.com). This method has been typically applied to isolated nuclei (Kotogány et al., 2010) but our earlier assessments demonstrated that isolated cell protoplasts are also suitable for two-parameter flow cytometry (Nosov et al., 2014).

### RNA isolation and quantitative RT-PCR (qRT-PCR) analysis

Total RNA was isolated from 100 mg of cells using a Spectrum Plant Total RNA Kit (Sigma) according to the manufacturer's manual. Synthesis of cDNA was performed using 500 ng of total RNA with Oligo(dT)_20_ primer (Invitrogen) and SuperScript III First-Strand Synthesis System following manufacturer's instructions (Invitrogen).

Amplification mixture (in a total reaction volume of 25 μl) contained 5 ng template cDNA, 5 × qPCRmix-HS SYBR (5 μl) (Evrogen, Moscow, Russian Federation) and 200 nM forward and reverse primers. All the primers used for qRT-PCR are listed in the Table 1. Reactions were run on CFX96 Touch (Bio-Rad, Hercules, California, USA) under the following cycling conditions: 95°C for 3 min, 45 cycles at 95°C for 10 s and 63°C for 30 s followed by melting curve analysis.

**Table 1:** **Sequences of oligonucleotide primers used for qRT-PCR analysis**.

**Gene name**	**Gene ID**	**Forward or reverse primer (5′–3′) (F or R)**	**Fragment size, bp**
*AtCDKA;1*	At3g48750.1	(F) ACTGACACTACATCCGATCG	140
		(R) GTGCCTTATAAACCACACCG	
*AtCDKB2;1*	At1g76540.1	(F) CTGGCAAGAACATTCCAACC	142
		(R) AGCCTCATTGTCTTGGGATC	
*AtCYCA2;3*	At1g15570.1	(F) AAGAGCCACTGGACCCAAC	136
		(R) ACTCGCCAATCCATGACCG	
*AtCYCB1;1*	At4g37490.1	(F) CCCAAAGAACAACGAACCGG	138
		(R) CCAGCCACTTTCTTCGGCT	
*AtCYCD3;1*	At4g34160.1	(F) CCTCCCATCAGTAGTTGCC	164
		(R) TGCGGTCCACTGGTAGTTG	
*AtACT2*	At3g18780.2	(F) CTCCTTGTACGCCAGTGGTC	111
		(R) CGGAGGATGGCATGAGGAAG	
*AtUBQ10*	At4g05320.2	(F) CCGTGATCAAGATGCAGATC	120
		(R) GAATGCCCTCCTTATCCTGG	

The Cq value was calculated from three independent biological experiments, each with four PCR replicates. At*ACT2* (At3g18780.2) and At*UBQ10* (At4g05320.2) were used as reference genes for data normalization as described by Pfaffl (2001) and Vandesompele et al. (2002).

### Statistical analyses

Data were subjected to analysis of variance using Minitab v.14 (Minitab Ltd, Coventry, UK), after which residual plots were inspected to confirm that data conformed to normality. Comparisons of data points from different treatments with controls were performed using Tukey multiple pairwise comparison test. Differences with *P* < 0.05 were considered significant.

## Results

### Ethylene and NO production during the cell suspension sub-cultivation cycle

**Figure 1:**
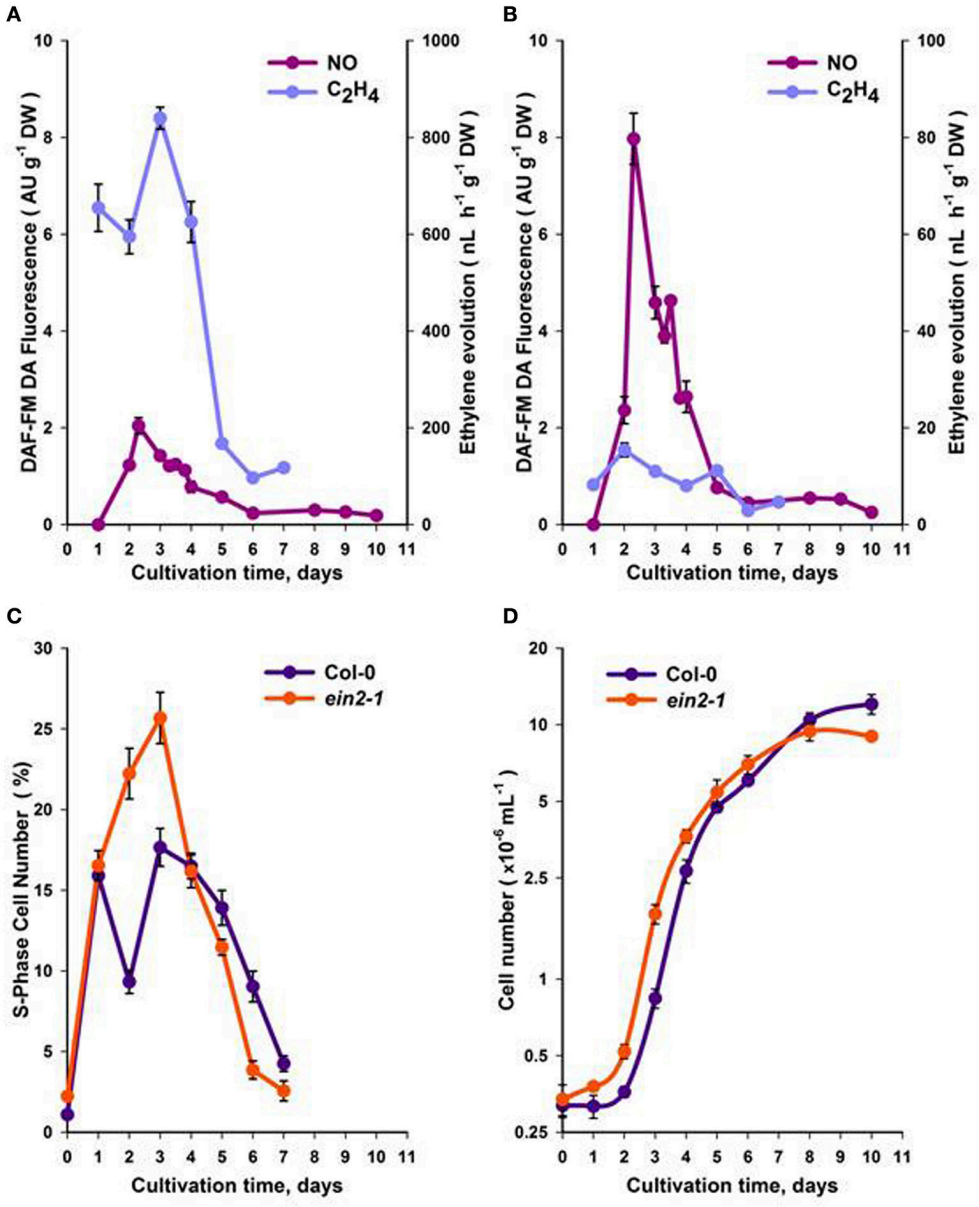
**Production of NO and ethylene within a sub-cultivation period of Col-0 (A)** and *ein2-1* **(B)** cultures. The portion of S-phase cells **(C)** and the total cell number **(D)**. On **(D)**, the ordinate axis is represented in a log scale. All values (means ± SE) are the averages of three independent biological experiments with five analytical replicates.

Using actively growing *Arabidopsis* Col-0 and *ein2-1* suspension cells (Figure S1), we studied the roles of ethylene and NO in CC progression. Initial experiments focused on observing the patterns of ethylene and NO generation over the sub-cultivation period. Measurements of ethylene using gas chromatography indicated that maximum ethylene production occurred over the first 5 days following sub-cultivation in both Col-0 (Figure 1A) and *ein2-1* (Figure 1B) cell lines although in the latter case levels were very low. To ensure that the observed patterns of ethylene production did not reflect hypoxic culture conditions, which would affect ACC oxidase (ACO) enzyme activity, O_2_ content was measured. Gas absorption chromatography of Col-0 and *ein2-1* cells in Suba Seal closed vials indicated that O_2_ levels did not drop lower than 20% which would allow ACO function and ethylene production.

NO production was evaluated within the same sub-cultivation cycle by measuring fluorescence from the NO-specific DAF-FM DA dye. Fluorescence was localized mainly in the cytoplasm and was most pronounced around the nuclei [Figure S2 (video)]. In Col-0 cells, maximum NO production occurred over the first 2–4 days following sub-cultivation (Figure 1A), while in *ein2-1* cell suspensions NO production was much higher compared to Col-0 over the same period (Figure 1B).

In Col-0 and *ein2-1* cells, ethylene and NO production over the cell sub-cultivation period were compared with cell division. Although both genotypes exhibited similarly shaped curves (Figures 1C,D), within 3–4 days of culturing Col-0 cells displayed fewer S-phase cells compared to *ein2-1* but after the 5th day rather more S-phase cells were observed (Figure 1C). This correlated with initially reduced levels of Col-0 cells (to 6 days) but with more cells at 10 days in comparison to *ein2-1* cells (Figure 1D).

### Assessing the effects of NO on suspension cell cultures

To assess the effects of NO, cells were treated with the NO-donor, SNP at the day 4 following sub-cultivation, which corresponded to some deceleration of the logarithmic growth phase (Figure 1D) when both Col-0 and *ein2-1* cultures retained similar numbers of S-phase cells (Figure 1C).

**Figure 2:**
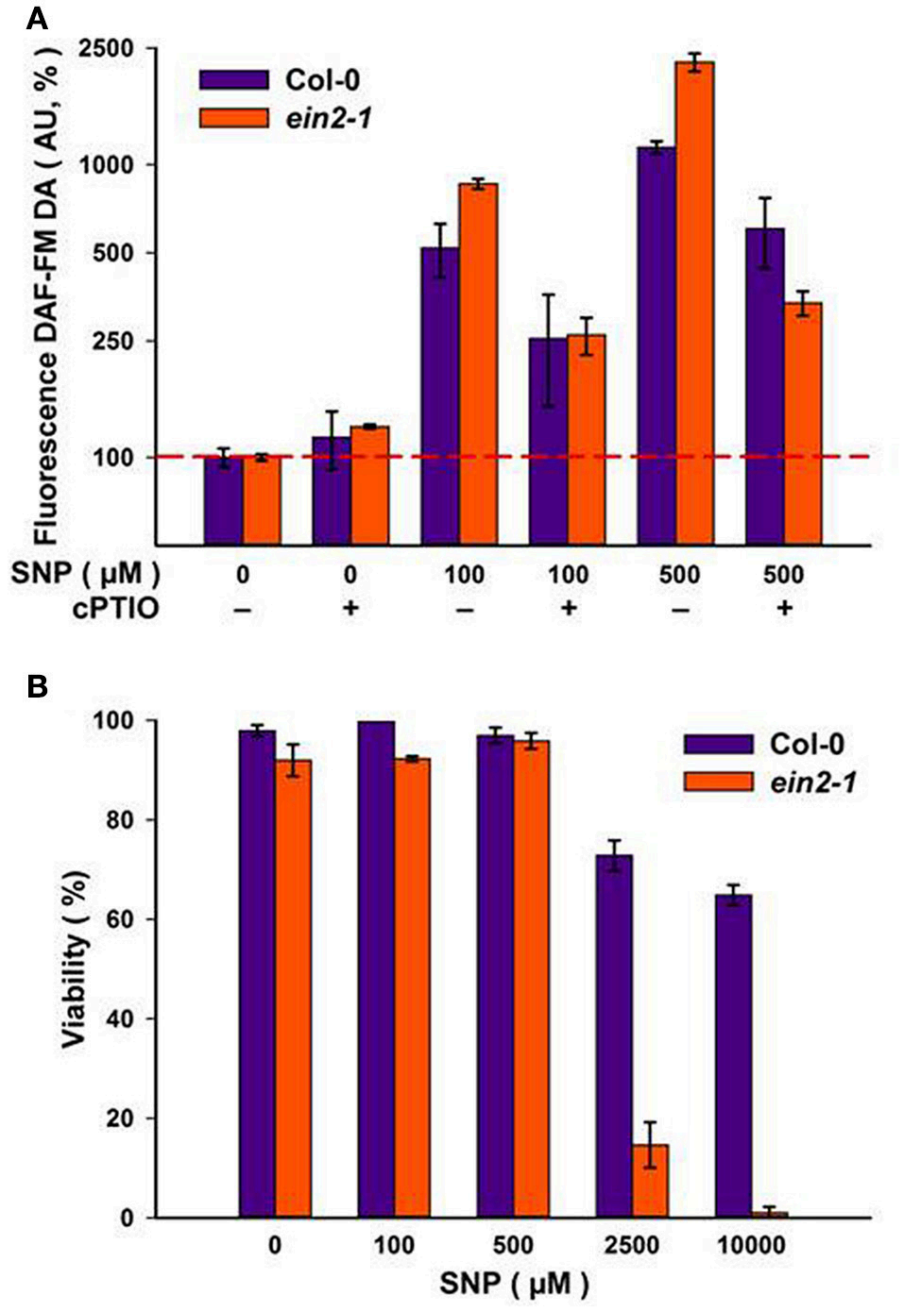
**Levels of NO as detected by DAF-FM DA fluorescence at the day four of sub-cultivation of Col-0 and *ein2-1* cells after 3-h treatment with sodium nitroprusside (SNP)**. The specificity of the fluorescent signal is demonstrated by parallel treatments with 100 μM of the NO scavenger cPTIO **(A)**. Cell viability was detected using Erythrosin B **(B)**. On **(A)**, the ordinate axis is represented on a log scale. Values are expressed as mean ± SE of biological triplicates with five analytical replicates.

Addition of increasing concentrations of SNP to Col-0 and *ein2-1* cells resulted in corresponding increases in NO production as reported by DAF-FM DA fluorescence (Figure 2A). The specificity of the fluorescence signal was established by its reduction when SNP was co-applied with 100 μM of the NO scavenger, cPTIO (Figure 2A). NO has also been linked to the initiation of cell death (Mur et al., 2008) but neither culture exhibited any significant loss in viability at SNP concentrations up to 500 μM (Figure 2B). Col-0 cells retained 60–70% viability at even higher SNP concentrations, whereas *ein2-1* cells exhibited considerable levels of cell death (Figure 2B). These results defined the range of SNP concentrations used in the further experiments.

To assess the impact of SNP on S-phase cells, flow cytometry after incorporation of the thymidine analog, EdU into DNA was used. Both suspension cultures were treated with either exhausted SNP or freshly prepared SNP solutions at concentrations of 5, 10, 20, 100, and 500 μM for 6 h at day 4 of sub-cultivation. EdU was added 1 h before the end of incubation with SNP and detected in the Click-iT reaction with the azide of Alexa Fluor 488.

**Figure 3:**
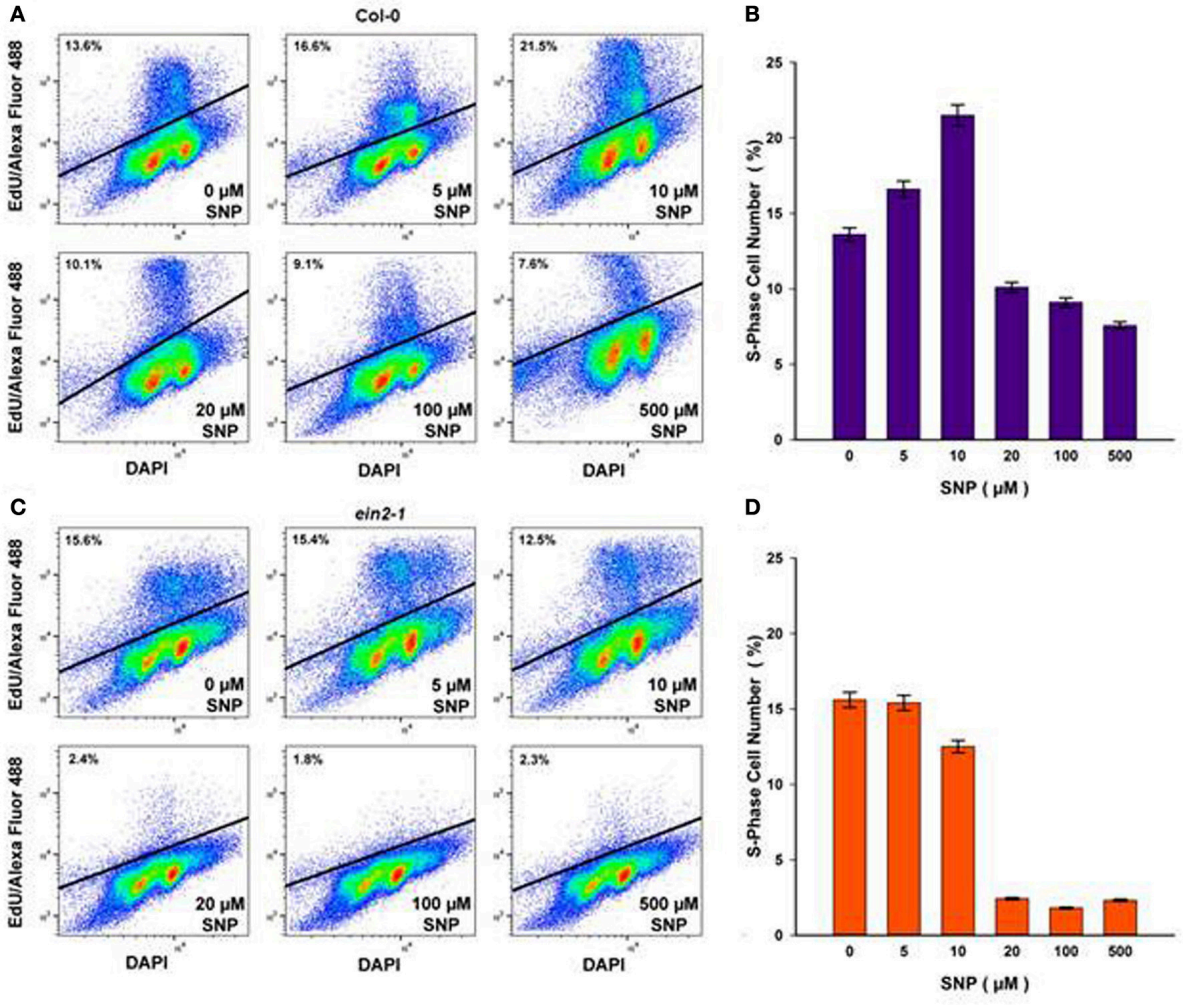
**S-phase cells (%) in Col-0 (A,B)** and *ein2-1* **(C,D)** after 6-h treatment with SNP as detected by flow cytometry after incorporation of 5-ethynyl-2′-deoxyuridine (EdU) into DNA and the Click-iT reaction with azide of Alexa Fluor 488. Representative bivariate plots of DNA content (based on DAPI fluorescence) and EdU incorporation (based on Alexa fluor) are shown in **(A)** and **(C)**. Above the solid line is a cluster of protoplasts having tag EdU–Alexa Fluor 488 (S-phase cells). Values in **(B)** and **(D)** are means ± SE of three independent biological experiments.

In Col-0 cultures, a 6-h treatment with SNP at 5 and 10 μM led to an increase in the number of S-phase cells from 13.6% in untreated cells to 16.6 and 21.5% in SNP-treated cells, respectively (Figures 3A,B). When the concentration of the SNP was increased to 20, 100, and 500 μM, the proportion of S-phase cells decreased significantly to 10.1, 9.1, and 7.6, respectively. This decrease was not linked to any effect on cell viability (Figure 2B).

In *ein2-1* cells, low SNP concentrations (5 and 10 μM) did not seriously affect the number of S-phase cells as compared to untreated cells (Figures 3C,D), however, when *ein2-1* cells were treated with higher concentrations of SNP (20, 100, and 500 μM), a dramatic fall in the number of S-phase cells was observed (Figures 3C,D).

The assessment of SNP effects on ethylene production in cultured cells of both genotypes revealed that it was repressed in Col-0 cells at all concentrations tested but was mostly unchanged in *ein2-1* cells (Table 2). Additionally, the *ein2-1* cultured cells showed a higher production of reactive oxygen and nitrogen species compared to Col-0 cells (Table 3).

**Table 2:** **Sodium nitroprusside (SNP) effect on ethylene evolution by Col-0 and *ein2-1* cultured cells**.

**SNP, μM**	**Ethylene evolution, nL g^−1^ FW h^−1^**	
	**Col-0**	***ein2-1***
0	54.6 ± 0.3	0.65 ± 0.2
20	46.3 ± 1.6	0.65 ± 0.07
100	28.7 ± 0.3	0.84 ± 0.14
500	25.8 ± 2.1	0.85 ± 0.22

**Table 3:** **Reactive oxygen (ROS) and nitrogen species (RNS) production in *Arabidopsis* cultured cells of wild type (Col-0) and the ethylene-insensitive mutant *ein2-1***.

**ROS/RNS**	**Col-0**	***ein2-1***
Superoxide	121.0 ± 13.4	189.3 ± 16.6
Peroxynitrite	125.3 ± 8.8	295.5 ± 16.1
ROS_*in*_	127.7 ± 5.7	382.5 ± 9.2

*At day 3 of cultivation, Col-0 and ein2-1 cells were sampled and ROS/NOS levels were estimated by measurement of fluorescence intensity with corresponding dyes. Superoxide was detected with dihydroethidium (DHE), peroxynitrite—with aminophenyl fluorescein (APF), intracellular ROS (ROS_in_)—with dichlorodihydrofluorescein diacetate (DCFH-DA). Values calculated as fluorescence units/1 μg of protein are means of three independent experiments with five replicates ± SD (standard deviations)*.

### NO and EIN2 effects on the expression of cyclin-dependent kinases and cyclins

**Figure 4:**
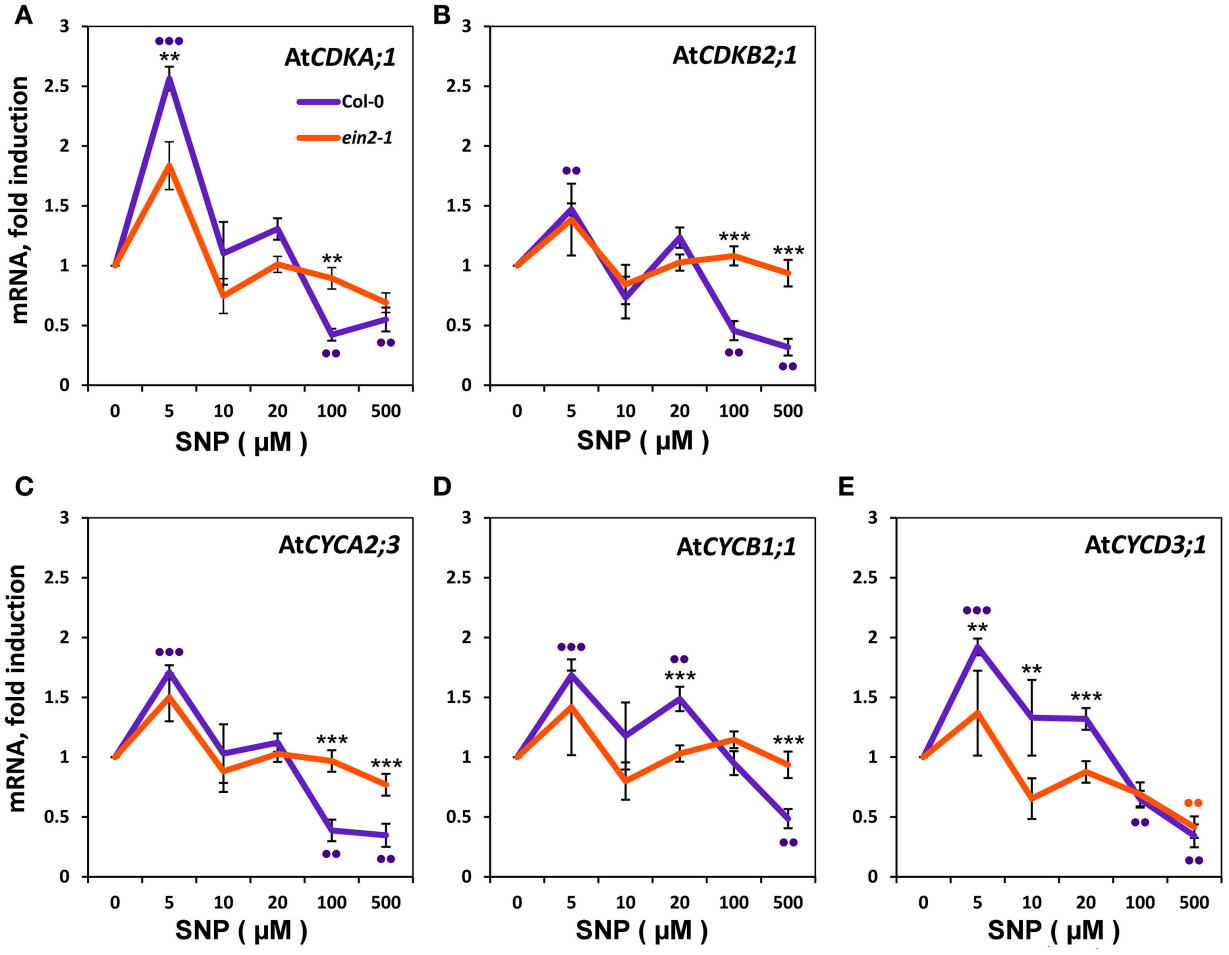
**Expression of Cyclins (*CYC*) and Cyclin-Dependent Kinases (*CDK*) in Col-0 and *ein2-1* cells after 6-h treatment with SNP**. mRNA levels in cultured Col-0 and *ein2-1* cells were examined by qRT-PCR for *CDKA;1* **(A)**, *CDKB2;1* **(B)**, *CYCA2;3***(C)**, *CYCB1;1* **(D)**, and *CYCD3;1* **(E)**. For qRT-PCR analysis, At*ACT2* and At*UBQ10* were used as the reference genes. Bars indicate standard errors of four technical replicates on biological triplicates. Asterisks indicate significant changes between Col-0 and *ein2-1* at a given SNP concentration. ^***^ *P* ≤ 0.001; ^**^ *P* ≤ 0.01. Dots indicate significant changes between untreated and SNP-treated Col-0 and *ein2-1* cells. ••• *P* ≤ 0.001; •• *P* ≤ 0.01.

To define the molecular events underlying the impact of NO on the transition of CC checkpoints, we examined the effect of SNP on the expression of genes for some *CDK*s and C*YC*s (Figure 4). Addition of 5 μM SNP to Col-0 cultures induce significant increases (*P* < 0.001) with *CDKA;1* (Figure 4A) and *CYCD3;1* (Figure 4E) that were ≥ two fold compared to controls. With *CDKB2;1, CYCA2;3*, and *CYCB1;1* there were significant increases with 5 μM SNP but were < two fold compared to untreated controls (Figures 4B,C,D). In the Col-0 cultures, addition of SNP at 100 and 500 μM SNP reduced the proportion of S-phase cells (Figures 3A,B) and this was accompanied by the significant (*P* < 0.01) reduction in the expression of *CYCA2;3, CYCD3;1, CDKA;1, CDKB2;1* (Figure 4), whereas the transcription of mitotic cyclin *CYCB1;1* was significantly reduced only at 500 μM SNP (Figure 4D). There were no significant changes in *CDKs* and *CYC*s gene expression compared to untreated controls with *ein2-1* at any SNP concentration with exception in *CYCD3;1* at 500 μM SNP (Figure 4E). Figure 4 also compares the response of Col-0 and *ein2-1* to SNP and highlight (with the exception of *CYCD3;1*) the abolition of a significant change in analyzed genes expression in *ein2-1* at > 10 μM SNP.

## Discussion

In animal cells, the role of NO in perturbing G1/S transitions of CC has been extensively characterized (Cui et al., 2005; Kumar et al., 2010; Napoli et al., 2013). We here demonstrate similar effects of NO on *Arabidopsis* cultured cells in a NO concentration dependent manner. Crucially, we have used the *ein2-1* mutant to demonstrate a key interaction with ethylene in this regulatory mechanism.

### NO and ethylene signaling events influence CC progression

Any consideration of the roles of NO and ethylene in the CC must be based on measurements of when these signals are produced. To provide a focus on ethylene effects our experimental strategy was based on comparing NO effects on Col-0 and *ein2-1* suspension cultures. Thus, we observed that NO and ethylene were maximally produced during an overlapping period of sub-cultivation. Crucially, the effect of SNP on Col-0 cells suggested that NO could repress rather than initiate ethylene production in Col-0 cells (Table 2). This was in contrast to the hypersensitive response during plant-pathogen interactions, where NO was required for ethylene production (Mur et al., 2008). However, the effect of SNP was consistent with the observed NO-dependent post-translational modification of methionine adenosyltransferase 1 (MAT1) to lower enzyme activity thereby decreasing ethylene synthesis (Lindermayr et al., 2006). Further levels of subtlety in this interaction are revealed by examination of responses in the *ein2-1* cultures. These data suggested that when ethylene production was suppressed that of NO was augmented (Figure 1). This implicates EIN2 as a regulatory switch influencing the CC patterns of both NO and ethylene production. Nonetheless, even in the absence of functionally active EIN2 (the case of *ein2-1* cells) the CC appeared to operate normally (Figures 1C,D). This could suggest that NO and ethylene production during the CC was irrelevant to the latter's control. However, some impacts on S-phase number and total cell number (Figures 1C,D) were noted. Thus, the relative rates of NO and ethylene production in both Col-0 and *ein2-1* cells suggest that these may be essential for the proper progression through the CC. Further, as a recent study showed that *Arabidopsis* ERFs, including ERF1 were required for the increase in cambial cell division (Etchells et al., 2012), it is appropriate to note our data relating to *ERF1* expression (Figure S3). In cultured *ein2-1* cells, where the ethylene signal transduction pathway is thought to be abolished (Alonso et al., 1999), we observed a high expression level of *ERF1*, which is a specific indicator of early ethylene signaling (Lorenzo et al., 2003). Taking into account that cultured *ein2-1* cells do not demonstrate reductions in cell number (Figure 1D) differences in *ERF1* expression could be the result of reduced ethylene signaling. This could suggest that in the absence of functionally active EIN2 alternative ethylene signaling pathway(s), which is likely to require a low level of ethylene, can operate (Cho and Yoo, 2015).

### Ethylene and NO act at the G1/S checkpoint in the CC

Typically, synchronized cells are used to study CC regulation, and aphidicolin is the most common chemical employed for culture synchronization. However, aphidicolin affects DNA polymerases and thus DNA replication blocked CC at the G1/S-phase boundary. This situation can be released by washing with fresh media in order to permit cells to move into the succeeding phase of the CC, but we have also shown a sharp increase in ethylene production within an hour after inoculation of cells into the fresh medium (Fomenkov et al., 2015). Therefore, the use of aphidicolin could complicates any dissection of ethylene effects on the CC. To avoid these problems as well as to minimize the mechanical manipulations with cells prior SNP treatments we used asynchronous Col-0 and *ein2-1* cultures in order to dissect the roles of ethylene, NO and EIN2 on cell proliferation.

The influence of NO on S-phase cells was estimated by flow cytometry. In Col-0, rather complex NO effects on S-phase were revealed. Low (5 and 10 μM) concentrations of SNP caused S-phase progression (Figures 3A,B), while high concentrations (20, 100, and 500 μM) caused a decrease in the proportion of S-phase cells. This was consistent with the results obtained on animal cells, where low NO concentrations (pM-nM) seemed to favor cell proliferation (Thomas et al., 2008). This may suggest a relative insensitivity to NO in our cultured plant cells (Figures 3A,B) but this was in agreement with those obtained for intact plants (Ötvös et al., 2005). Moreover, it should be kept in mind that SNP does not release all of its NO instantaneously (Mur et al., 2013) and the actual dose of NO obtained by cells from the surrounding medium could be lower than the concentration of SNP which had been used for treatments.

A halt in the CC progression could be associated with cell differentiation which can be preceded by endoreduplication (De Veylder et al., 2007). However, an increase in endoreduplication was not observed in either Col-0 or *ein2-1* cells after 6-h treatment with high NO concentrations (data not shown). Currently, it is unclear how far this finding reflected an effect of exogenous NO at the highly reduced level of endogenous ethylene (Figure 1B). It is worth noting that there is a report that high SNP concentrations contributed to endoreduplication in the *chlorophyll a/b binding protein under-expressed* (*cue1*) mutant, which produces more NO than wild type and, moreover, is NO supersensitive (Bai et al., 2012).

Our definition of the molecular events underlying NO/ethylene effect on the CC naturally focused on the expression pattern of *CYC*s and *CDK*s. CYCs of the D-type (CYCDs) are CDKA;1 partners which control the G1/S transition (Menges et al., 2006; de Jager et al., 2009). CYCA2;3 is a marker of G2/M transition, and CYCB1;1 is a mitotic CYC. CDKB1;1 operates with CYCA2;3 and CDKB2;1/CDKB2;2—with CYCB1;1 (Menges et al., 2005; Inzé and De Veylder, 2006; Van Leene et al., 2010; Komaki and Sugimoto, 2012). Referring to the results presented, we suggest that in Col-0 cells NO application at low concentrations positively influences CC progression at G1/S transition (Figures 3A,B) at least due to stimulation of *CDKA;1* and *CYCD3;1* expression (Figures 4A,E). It should be noted, that expression of *CYCA2;3, CYCB1;1*, and *CDKB2;1* operating at G2/M transition and in M-phase of CC barely increased (Figures 4C,D,B), further suggesting that low concentrations of NO stimulated G1/S transition. At the same time, in *ein2-1* cells, low concentrations of SNP had little effect on both the S-phase index (Figures 3C,D) and expression of the genes associated with the CC (Figure 4) compared to Col-0.

**Figure 5:**
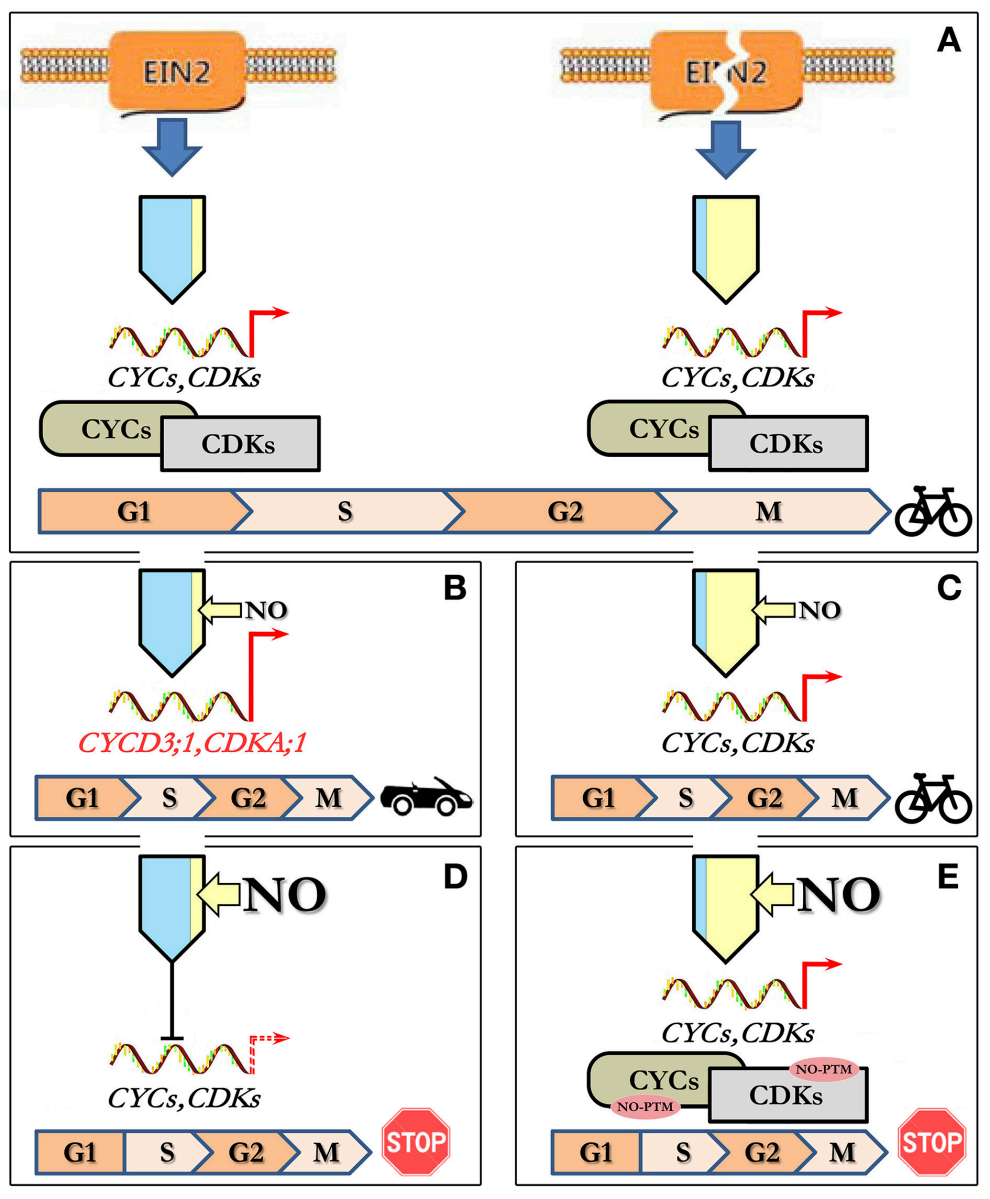
**Model describing concentration-dependent NO–ethylene interactions influencing CC progression. (A)** Under standard growth conditions, cultured Col-0 and *ein2-1* cells have opposite levels of ethylene (blue) and NO (yellow) production with a similar proliferation activity controlled by appropriate regulation of CDKs and CYCs. **(B)** Treatment of Col-0 cells with low NO concentrations (narrow yellow arrow) results in stimulation of G1-to-S phase transition due to increased expression of *CDKA;1* and *CYCD3;1*. **(C)** In *ein2-1* cells with high endogenous NO, low concentrations of NO supplied do not affect either the index of S-phase cells or the expression of *CDKs* and *CYCs* leading to unchanged CC progression. **(D)** At high levels of exogenous NO (wide yellow arrow), in Col-0 cells, the expression of CC-related genes is reduced and concomitantly CC progression slows down. **(E)** Treatment of *ein2-1* cells with high NO concentrations on the background of the high endogenous NO level have no significant effect on the expression of CC-associated genes but a sharp reduce in the number of S-phase cells indicates a stop in G1/S transition most likely due to NO-dependent post-translational modifications (NO-PTM) of the CC-related proteins.

Based on the above observations, it is possible to suggest a tentative model for NO/ethylene effects on the CC of *Arabidopsis* cultured cells (Figure 5). Under standard cultivation conditions, cells of Col-0 (with functional EIN2) and *ein2-1* (with “broken” EIN2) have opposite levels of ethylene and NO production but almost the same levels of proliferation which should be controlled by appropriate transcription/translation of CDKs and CYCs (Figure 5A). High levels of endogenous NO apparently inhibit ethylene production in *ein2-1* cells as exogenous NO does in Col-0 cells. Low level of endogenous ethylene in *ein2-1* cells likely provides the cell proliferation control bypassing EIN2. Treatment of Col-0 cells with low concentrations of NO (5 and 10 μM of SNP) on the background of low endogenous NO level stimulates cell transition into S-phase of CC due to increased expression of associated *CDKA;1* and *CYCD3;1* (Figure 5B). In *ein2-1* cells with high endogenous NO level, the low concentrations of NO supplied (5 and 10 μM of SNP) do not affect both the index of S-phase cells and expression of *CDKs* and *CYCs* tested. Under such circumstances CC progression remains unchanged (Figure 5C). In Col-0 cells, high levels of exogenous NO decrease the expression of *CDKA;1, CDKB2;1*, and *CYCA2;3, CYCB1;1, CYCD3;1* resulting in downregulation of CC progression (Figure 5D). Finally, the treatments of *ein2-1* cells with high concentrations of NO on the background of the high endogenous NO level have no significant effect on the expression of the CC-associated genes but rapidly reduce the number of S-phase cells indicating that G1/S transition comes to a stop (Figure 5E). Thus, from the above appears that CDKA;1 is the main response to low (5 μM) SNP and EIN2 is required for full responses at each SNP concentration. Additionally or alternatively, the CC progression might be mediated by NO-dependent post-translational protein modifications including those involved in CC regulation (Figure 5E) since *ein2-1* cultured cells accumulate substantial amounts of both ROS and RNS (Table 3). Clearly, such a simple model requires further characterization but it does generate several hypotheses on which further studies can be based.

To our knowledge, this is the first report demonstrating EIN2-dependent NO-provoked CC regulation in cultured plant cells. The data presented here point to the similarity of NO effects on the CC progression in cells of whole plants and *in vitro* cultured plant cells and, moreover, show the similarity of these effects to those in animal cells. Perhaps, most importantly, the NO-dependent effects on the CC progression, which were revealed in our work, provide a possible link to the functioning of ethylene signaling pathway(s).

## Author contributions

GN Designed and supervised most of the experiments and contributed to the writing of the paper; LM Contributed to the design of the experiments and the writing of the paper; AN Contributed to the design of the experiments and the writing of the paper. Contributed to the drawing up of the integrated model presented in Figure 5; AF provided technical support in maintaining the cell cultures and drawing up figures presented; KM undertook the qRT-PCR experiments; AM undertook all NO donor treatments and flow-cytometry based experiments; ES designed and supervised the flow-cytometry based experiments; VR undertook ethylene measurements; MH was involved in discussion on experimental design and contributed to the writing of the paper.

### Conflict of interest statement

The authors declare that the research was conducted in the absence of any commercial or financial relationships that could be construed as a potential conflict of interest.

## Supplementary material

Figure S1 **Microphotographs of *Arabidopsis thaliana* cultured cells of wild type (Col-0) and ethylene-insensitive mutant *ein2-1***. Bar scales are 20 μm.

Figure S2 (video) **NO visualization within *Arabidopsis thaliana* (Col-0) cells represented as sequential series of the cell's optical sections stained with DAF-FM DA (Axio Imager Z2 with ApoTome.2 function)**.

Figure S3 **Expression of *ERF1* (At4g20550) in Col-0 and *ein2-1* cultured cells at day two of sub-cultivation as detected using RT-PCR**. Primers used for amplification were (F) TTCAGTCCCCATTCTCCGGC, (R) GCCGTCGTCTTACGCCTCTG. The numbers under the bands indicate the expression of *ERF1* relative to At*UBQ10* expression used as the reference gene. Arrow indicates the fragment size (bp).
