# Supplementary figures and images for: Nitric Oxide Has a Concentration-Dependent Effect on the Cell Cycle Acting via EIN2 in Arabidopsis thaliana Cultured Cells
Source: Front Physiol. 2017 Mar 10;8:142. doi: 10.3389/fphys.2017.00142 (PMC5344996; doi:10.3389/fphys.2017.00142)

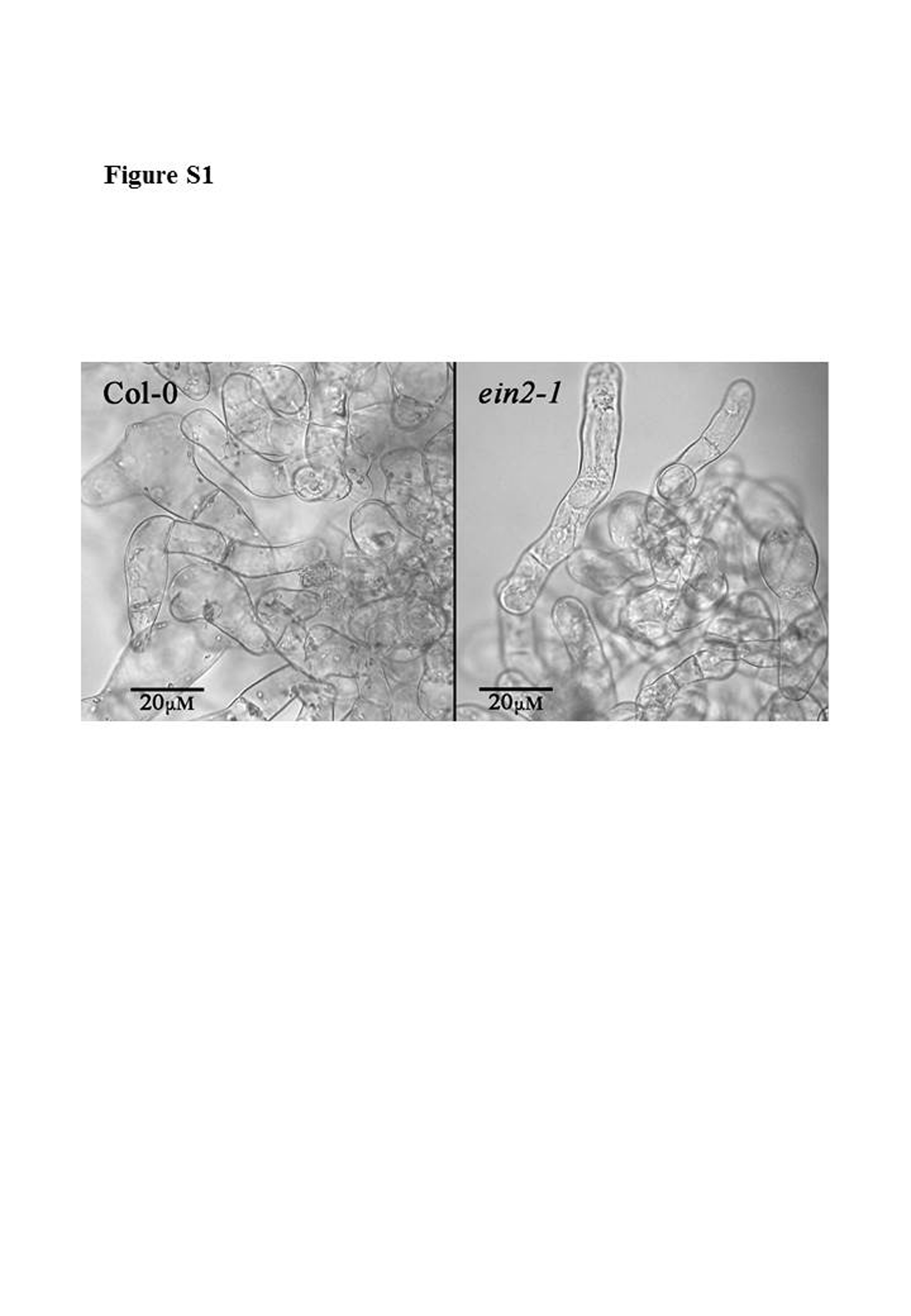

Supplement: Figure S1 — Microphotographs of Arabidopsis thaliana cultured cells of wild type (Col-0) and ethylene-insensitive mutant ein2-1. Bar scales are 20 μm. [file Image1.JPEG]

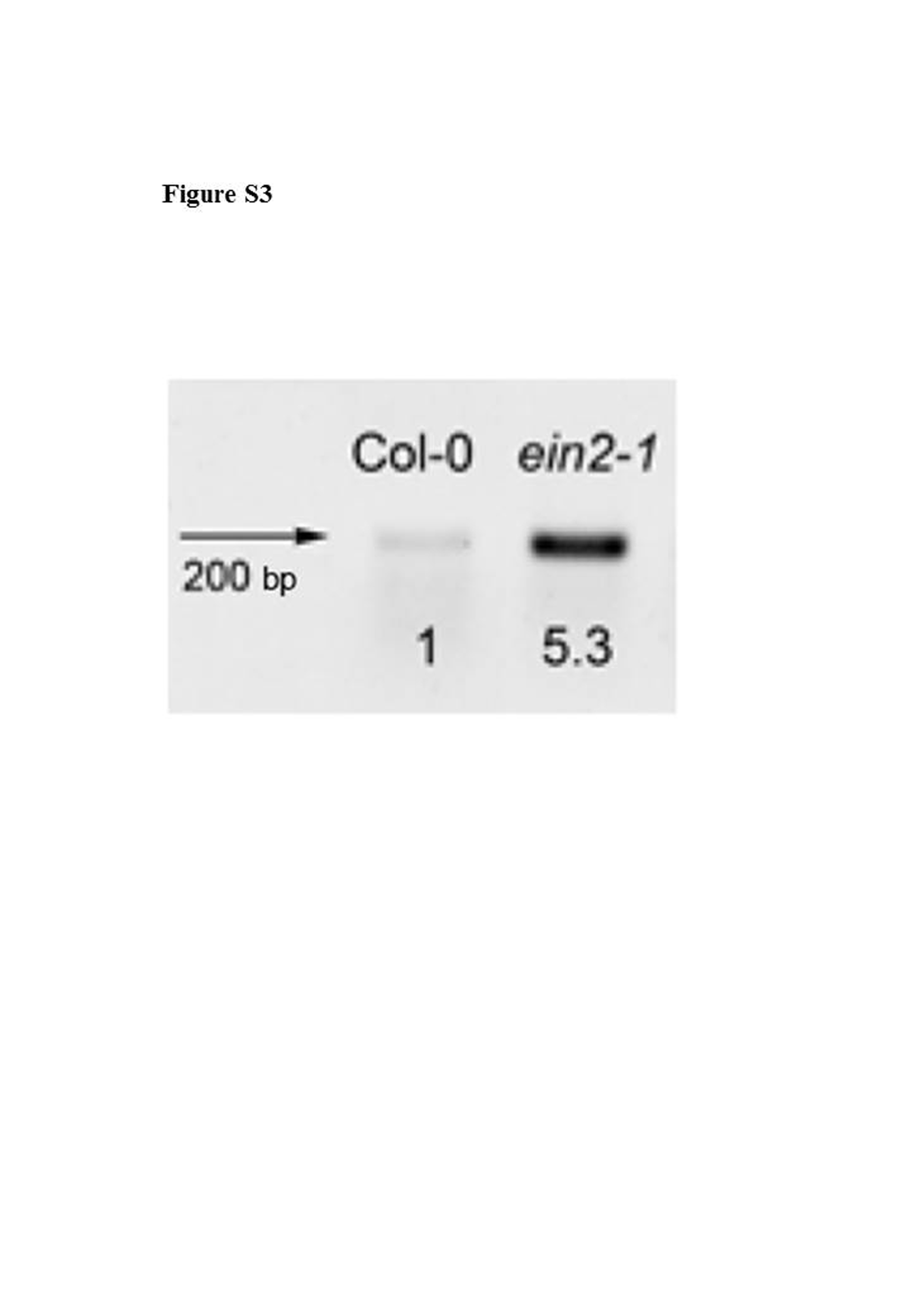

Supplement: Figure S3 — Expression of ERF1 (At4g20550) in Col-0 and ein2-1 cultured cells at day two of sub-cultivation as detected using RT-PCR. Primers used for amplification were (F) TTCAGTCCCCATTCTCCGGC, (R) GCCGTCGTCTTACGCCTCTG. The numbers under the bands indicate the expression of ERF1 relative to AtUBQ10 expression used as the reference gene. Arrow indicates the fragment size (bp). [file Image3.jpeg]
